# Adenocarcinoma arising from chronic perianal crohn's disease: a case report

**DOI:** 10.11604/pamj.2015.22.140.7875

**Published:** 2015-10-14

**Authors:** Hanane Massit, Meryem Edderai, Rachida Saouab, Hassan Seddik, Jamal El Fenni, Ahmed Benkirane

**Affiliations:** 1 Department of Gastroenterology II, Mohamed V Teaching Military Hospital, Mohamed V-Souissi University, Rabat, Morocco; 2 Department of Radiology, Mohamed V Teaching Military Hospital, Mohamed V-Souissi University, Rabat, Morocco

**Keywords:** Crohn′s disease, perianal disease, perineal adenocarcinoma

## Abstract

Malignant transformation of perineal fistula in Crohn's disease has rarely been reported. We report a case of Crohn's disease with recurrent perineal fistulas. A 36-year-old male, diagnosed with Crohn's disease at the age of 24, developed adenocarcinoma in an anorectal fistula that had existed for years. He was treated with adjuvant chemoradiotherapy but died. A high index of suspicion and regular surveillance is recommended in chronic anorectal fistulas in Crohn's disease. The shorter duration of Crohn's fistulas prior to malignant degeneration necessitates an aggressive approach to rule out cancer.

## Introduction

The risk of malignancy should be seriously considered in the management of Crohn′s disease, especially in young patients. Although perianal fistulas are common in the natural history of Crohn's disease, accounting for around 30-50% of cases, its malignant transformation is extremely rare, from 0.004 to 0.7% [1]. The etiology of fistula related cancer remains a subject of debate. Probably chronic irritation at either end of a fistula can trigger the degeneration of scar tissue into cancer. Alternatively, the carcinoma may be the cause of the fistula.

## Patient and observation

**Figure 1:**
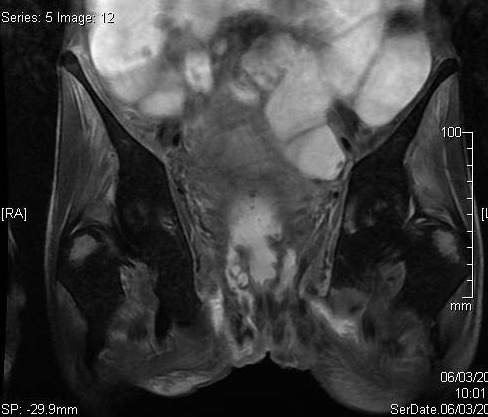
Coronal T2 W fat-sat images showing multiples supra sphincteric fistula, with secondary tracts in ischio anal and gluteal regions

**Figure 2:**
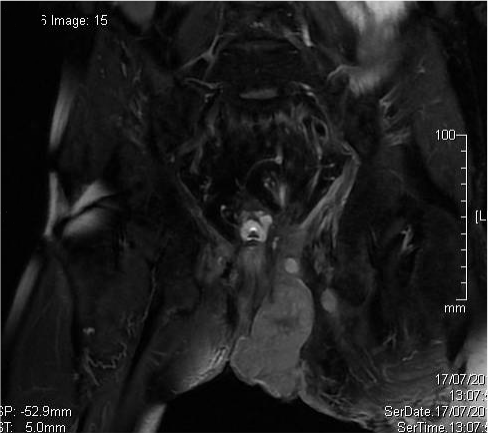
Dynamic postcontrast fat-suppressed coronal T1-weighted image: Lobulated left sided perineal mass measuring about 7 cm connected to the anal canal, with mild heterogeneous enhancement, it's associated with perineal fat infiltration and left side gluteal abcess

We present the case of a 36-year-old male diagnosed with severe active fistulising Crohn′s disease (mean duration 12 years) whose disease has not responded to conventional therapy, including antibiotics, drainage immunosuppressive treatments and Infliximab, with long-lasting and difficult to manage perianal and intestinal disease. The diagnosis of Crohn′s disease was established by clinical, radiological, endoscopic and pathological features. MRI showed multiples supra sphincteric fistula, with secondary tracts in ischio anal and gluteal regions (Figure 1). This patient has been reasonably well despite grossly distorted anatomy until he reported increasing anorectal symptoms three to six months before the diagnosis of malignancy. Due to the seriousness of perianal disease, an MRI was performed: Lobulated left sided perineal mass measuring about 7 cm connected to the anal canal, with mild heterogeneous enhancement, it′s associated with perineal fat infiltration and left side gluteal abcess (Figure 2). The diagnosis of malignancy was established at examination under anaesthetic and by histological examination of the excised anal skin tag. The histological piece showed moderately differentiated rectal and perianal adenocarcinoma, implanted in a deep perianal fistula. The tumour was inoperable. The patient received chemoradiotherapy and has been followed up for only a short period and he died.

## Discussion

Carcinomas arising in longstanding anorectal Crohn′s disease represents a specific clinical problem, the histological distinction may have important therapeutic implications, as squamous cell carcinoma is more responsive to radiotherapy than adenocarcinoma. The possible reasons for the development of anorectal malignancy in Crohn′s disease include chronic inflammation and sepsis, immunosuppressive drug use, and viral infection [2]. This mainly occurs in patients with long-lasting disease [3], although the duration is shorter in women [4]. Many patients with anal lesions have few symptoms despite grossly distorted perianal anatomy. A recent change in symptoms may herald the onset of malignancy. In our case, our patient did have long-lasting and difficult to manage perianal disease, so constant inflammatory activity could have played an important role in the development of the tumor. The diagnosis is difficult and generally late, since the clinical manifestations are nonspecific or attributed to the typical symptoms of perianal disease [5]. Some authors [3] recommend annual routine examinations in patients with perianal disease of more than 10 years duration due to the rapid growth of the adenocarcinoma in a perianal fistula. Examination under anaesthetic is indicated if pain precludes normal clinical examination or if an anal biopsy is required. MRI showed close correlation with findings at examination under anaesthetic. If malignancy is suspected despite a negative examination and biopsy, the investigations should be repeated after a short period of observation. It is hoped tumours can be detected at an earlier and curable stage by this comparatively simple approach [2]. When malignancy is found, an aggressive surgical approach and complementary therapy are mandatory. Results after surgery are poor with high postoperative relapse. There is no clear evidence on the role of adjuvant chemoradiotherapy, although mucinous tumors have a worse response to this therapy and poorer survival [6].

## Conclusion

Adenocarcinoma implanted on a perianal fistula is an aggressive tumor, with a difficult and late diagnosis, conferring a poor prognosis. Physicians should have a high level of suspicion of cancer in patients with longstanding perianal Crohn's disease who have a change in symptoms. Any unusual or persistant perianal lesion should be biopsied. The shorter duration of Crohn's fistulas prior to malignant degeneration necessitates an aggressive approach to rule out cancer.
